# Assessment of funnel plot asymmetry and publication bias in reproductive health meta-analyses: an analytic survey

**DOI:** 10.1186/1742-4755-4-3

**Published:** 2007-04-16

**Authors:** João P Souza, Cynthia Pileggi, José G Cecatti

**Affiliations:** 1 Department of Obstetrics and Gynecology, School of Medical Sciences, University of Campinas, Brazil; 2 Clinical Epidemiology Collaborative Group, Women's Integrated Health Care Center, University of Campinas, Brazil

## Abstract

**Background:**

Despite efforts to assure high methodological standards, systematic reviews may be affected by publication bias. The objective of this study was to evaluate the occurrence of publication bias in a collection of high quality systematic reviews on reproductive health.

**Methods:**

Systematic reviews included in the Reproductive Health Library (RHL), issue No 9, were assessed. Funnel plot was used to assess meta-analyses containing 10 or more trials reporting a binary outcome. A funnel plot, the estimated number of missing studies and the adjusted combined effect size were obtained using the "trim and fill method". Meta-analyses results that were not considered to be robust due to a possible publication bias were submitted to a more detailed assessment.

**Results:**

A total of 21 systematic reviews were assessed. The number of trials comprising each one ranged from 10 to 83 (median = 13), totaling 379 trials, whose results have been summarized. None of the reviews had reported any evaluation of publication bias or funnel plot asymmetry. Some degree of asymmetry in funnel plots was observed in 18 of the 21 meta-analyses evaluated (85.7%), with the estimated number of missing studies ranging from 1 to 18 (median = 3). Only for three meta-analyses, the conclusion could not be considered robust due to a possible publication bias.

**Conclusion:**

Asymmetry is a frequent finding in funnel plots of meta-analyses in reproductive health, but according to the present evaluation, less than 15% of meta-analyses report conclusions that would not be considered robust. Publication bias and other sources of asymmetry in funnel plots should be systematically addressed by reproductive health meta-analysts. Next amendments in Cochrane systematic reviews should include this type of evaluation. Further studies regarding the evolution of effect size and publication bias over time in systematic reviews in reproductive health are needed.

## Background

Implementing best practices is a major goal in health services [1]. However, the identification of such practices depends on the evaluation and synthesis of a large amount of scientific information. This may be achieved by carrying out systematic reviews and meta-analyses, which have come to represent important sources of evidence-based knowledge for clinicians, policy makers and researchers [2].

A systematic review is an observational study of the scientific literature based on individual studies. It may contain meta-analyses, which are statistical procedures developed for summarizing effects across individual studies. The ideal meta-analysis should combine data appropriately to produce a more complete and meaningful estimate of the overall effect [3]. Nevertheless, despite efforts to assure high methodological standards, systematic reviews may be affected by publication bias, one of the major drawbacks of such studies and a threat to their validity. Publication bias occurs whenever the results of a set of published studies differ from the results of all the research performed on a specific topic [3]. A publication-biased meta-analysis may present an ineffective or unsafe intervention as being effective or safe, or not recommend an effective or safe intervention because the results of some studies already performed are not included. Furthermore, publication bias may be partially responsible for occasional discrepancies between the conclusions of previous meta-analyses and subsequent large multicenter trials [4].

The assessment of publication bias is a relatively new recommendation but reported in several relevant meta-analyses reporting guidelines [5-8]. However, it has been observed that only few meta-analyses have actually evaluated publication bias (3.2% – 6.5%) [9]. It is also unclear whether reproductive health meta-analyses are affected by publication bias, since we have been unable to identify any previous reports assessing publication bias and its effects on meta-analyses in reproductive health. The objective of this survey was, therefore, to evaluate the occurrence of publication bias in a collection of high quality systematic reviews on reproductive health.

## Methods

This is an analytic survey carried out to evaluate the impact of publication bias on the results of meta-analyses of reproductive health interventions. Systematic reviews included in the World Health Organization (WHO) Reproductive Health Library (RHL), issue No 9, were assessed [10]. The RHL is a WHO instrument for documenting and disseminating best practices in the field. It reproduces the most relevant Cochrane systematic reviews related to reproductive health, adding some practical aspects and pertinent comments for developing country settings, as well as implications for research [10].

There are several methods of assessing the occurrence of publication bias. A common approach is based on scatter plots of the treatment effect estimated by individual studies versus a measure of study size or precision (the "funnel plot"). In this graphical representation, larger and more precise studies are plotted at the top, near the combined effect size, while smaller and less precise studies will show a wider distribution below. If there is no publication bias, the studies would be expected to be symmetrically distributed on both sides of the combined effect size line. In case of publication bias, the funnel plot may be asymmetrical, since the absence of studies would distort the distribution on the scatter plot [3].

The "trim and fill" method examines the existence of asymmetry in the funnel plot and is recommended as a tool for the assessment of the robustness of the results of meta-analyses (sensitivity analysis). The method consists of a rank-based data augmentation procedure that statistically estimates the number and location of missing studies. The main application of this method is to adjust for the possible effects of missing studies [11]. If the conclusion of the meta-analysis remains unchanged following adjustment for the publication bias, the results can be considered reasonably robust, excluding publication bias.

In the present study, funnel plot asymmetry was used to assess meta-analyses containing 10 or more trials reporting a binary outcome. In each review, the meta-analysis with the greatest number of trials was selected for evaluation. Therefore those meta-analyses may not necessarily represent the primary outcomes for the review. If two or more meta-analyses had a similar number of trials, the one listed first in the review was selected [12]. To achieve consistency across meta-analyses, endpoints were re-coded if necessary so that an effect size below 1 always indicated a beneficial effect of the intervention.

The following data were extracted from each meta-analysis: study name, subgroups within the study, data on effect size, year of publication of the most recent trial included and assessment of publication bias. After extracting the data and compiling a database, statistical analysis was performed using the Comprehensive Meta-Analysis^® ^software program (version 2.2.034, USA, 2006). The database was checked twice for the presence of inconsistencies. For each meta-analysis, a funnel plot, the estimated number of missing studies and the adjusted combined effect size were obtained using the "trim and fill method". This procedure was applied to both sides of each funnel plot. The model of effect (fixed-effects or random-effects) used was the same as that applied in the primary meta-analysis. Results of "trim and fill method" were validated by using Stata (Stata Corporation, College Station, TX, USA)

In Cochrane systematic reviews, the Mantel-Haenszel method is usually applied to estimate relative risk by using the Review Manager (RevMan) software program [8]. In order to comply with the requirements of the Comprehensive Meta-Analyses^® ^software program used in the present study, values obtained for the adjustment for publication bias were converted through the inverse variance method. In case of possible publication bias, the meta-analyses were submitted to a more detailed assessment.

## Results

The 9^th ^issue of the WHO Reproductive Health Library included 105 Cochrane Systematic Reviews. A total of 21 systematic reviews contained meta-analyses with 10 or more trials reporting a binary outcome. In this set of meta-analyses, the number of trials comprising each one ranged from 10 to 83 (median = 13), totaling 379 trials, whose results have been summarized. None of the reviews had reported any evaluation of publication bias or funnel plot asymmetry. During data extraction, the endpoints of one meta-analysis were re-coded to guarantee consistency across meta-analyses [13].

The main characteristics of the selected meta-analyses and the adjustments performed are shown in Table 1. According to the "trim and fill method", some degree of asymmetry in funnel plots was observed in 18 of the 21 meta-analyses evaluated (85.7%). In meta-analyses in which asymmetric funnel plot was found, the estimated number of missing studies ranged from 1 to 18 (median = 3). All summary plots of meta-analyses, together with their respective "trimmed and filled" funnel plots, are shown in Appendix 1 [See Additional file 1].

**Table 1:** Principal characteristics of selected meta-analyses and the adjustment performed according to the "trim and fill method"

**Meta-analysis**	**Number of trials**	**Estimated number of missing studies**	**Model of Effect used**	**Original combined effect estimates**	**Adjusted effect estimates**	**Year of last trial included**
Hofmeyr ^18^	12	0	Fixed	0.35 (0.22–0.56)	Unchanged	1999
Smaill ^19^	83	18	Random	0.39 (0.35–0.43)	0.42 (0.37–0.47)	2001
Hopkins ^14^	14	7	Fixed	0.92 (0.70–1.23)	0.57 (0.45–0.73)*	1993
Hopkins ^20^	11	3	Fixed	1.08 (0.74–1.58)	0.96 (0.68–1.36)	1996
Smaill ^21^	13	0	Fixed	0.24 (0.19–0.32)	Unchanged	1987
Kenyon ^22^	13	1	Fixed	0.90 (0.74–1.10)	0.91 (0.74–1.11)	2001
McDonald ^23^	13	2	Fixed	0.87 (0.74–1.02)	0.92 (0.78–1.07)	2003
Abalos ^24^	22	1	Fixed	0.75 (0.47–1.19)	0.73 (0.46–1.15)	1995
Knight ^25^	37	14	Fixed	0.87 (0.80–0.94)	0.91 (0.84–0.99)	1999
King ^26^	11	1	Fixed	1.49 (0.67–3.34)	1.35 (0.62–2.95)	2002
Atallah ^27^	11	5	Random	0.35 (0.20–0.60)	0.50 (0.32–0.78)	2001
Martin-Hirsch ^13^	11	3	Fixed	0.28 (0.25–0.31)	0.27 (0.25–0.30)	1994
Thacker ^28^	10	5	Fixed	1.39 (1.21–1.59)	1.17 (1.04–1.33)	1993
Hodnett ^15^	15	5	Fixed	0.91 (0.83–0.99)	0.94 (0.86–1.03)*	2002
Cheng ^16^	10	1	Fixed	0.63 (0.44–0.92)	0.70 (0.49–1.00)*	2002
Crowley ^29^	19	3	Fixed	0.20 (0.06–0.70)	0.25 (0.08–0.73)	1992
Oates-Whitehead ^30^	14	0	Fixed	1.05 (0.83–1.34)	Unchanged	2002
Hodnett ^31^	13	2	Fixed	0.99 (0.90–1.09)	0.98 (0.89–1.08)	2001
Carroli ^32^	10	2	Fixed	0.81 (0.70–0.93)	0.83 (0.72–0.95)	1998
Johanson ^33^	12	2	Fixed	0.59 (0.51–0.68)	0.60 (0.52–0.69)	1996
Hofmeyr ^34^	25	7	Random	0.65 (0.58–0.73)	0.72 (0.64–0.81)	2003

* Conclusion is possibly affected by publication bias

In 18 of the 21 meta-analyses evaluated, the assessment procedure and the adjustments for publication bias had no effect on the conclusions (Table 1); however, in the remaining three (14.3%, 3:21), the conclusion cannot be considered robust due to a possible publication bias [14-16]. These results were obtained applying the same model of effect used in the primary meta-analysis (fixed-effects or random-effects), but were confirmed using the alternative method (data not shown).

**Figure 1:**
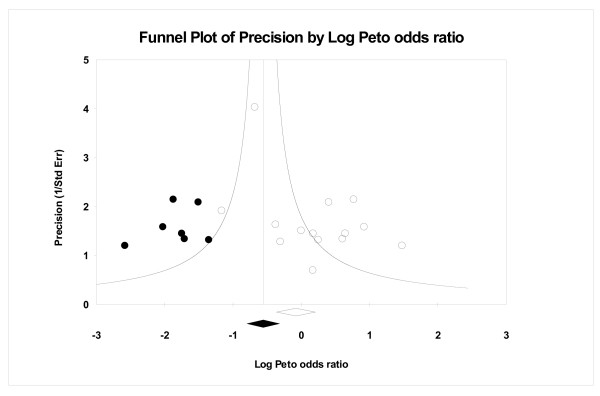
A filled funnel plot of the antibiotics prophylaxis regimens for cesarean section data, with filled circles denoting the imputed missing studies. The bottom diamonds show summary effect estimates before (open) and after (filled) publication bias adjustment.

**Figure 2:**
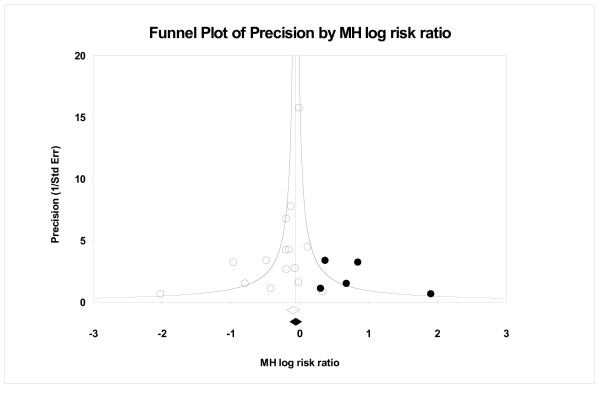
A filled funnel plot of the continuous support for women during childbirth data, with filled circles denoting the imputed missing studies. The bottom diamonds show summary effect estimates before (open) and after (filled) publication bias adjustment.

**Figure 3:**
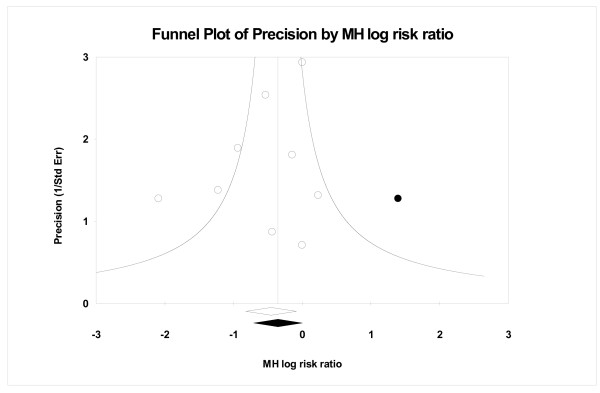
A filled funnel plot of the interventions for emergency contraception data, with filled circles denoting the imputed missing studies. The bottom diamonds show summary effect estimates before (open) and after (filled) publication bias adjustment.

Figures 1 and 2 show filled funnel plots with summary effect estimates before and after adjustment for the publication bias. Both meta-analyses included a similar number of trials and both presented asymmetric funnel plots of identified studies (open circles). After adjustment for the publication bias, the estimated number of missing studies entered into the funnel plot (filled circles) was moderate, 7 and 5 respectively. The summary of estimates obtained before (open diamond) and after the adjustment (filled diamond) indicates that, if really such a number of missing studies exists, the impact on the conclusion may not be negligible. Figure 3 also presents a filled funnel plot and, although the estimates suggest that only one study may be missing, the practical impact on the conclusion should be considered. Table 2 summarizes the practical impact of the adjustment for publication bias on conclusions in these three meta-analyses.

**Table 2:** The practical impact on conclusions of three meta-analyses submitted to publication bias adjustment.

**Authors**	**Topic**	**Comparison**	**Outcome**	**Non-adjusted meta-analysis conclusion (transcription)**	**Adjusted meta-analysis conclusion**
Hopkins ^14^	Antibiotic prophylaxis regimens for cesarean section	Any single dose of systemic antibiotic versus any multiple dose	Endometritis	"There is no evidence from this meta-analysis to recommend multiple doses of antibiotics"	Any single dose systemic regimen (pre, post or intra-operative) may be *more effective *than any multiple dose regimen
Hodnett ^15^	Continuous support for women during childbirth	Continuous one-to-one intrapartum support versus usual care	Cesarean birth	"Women who had continuous, one-to-one support during labour were less likely to have a caesarean birth"	Continuous support *may not reduce *the occurrence of cesarean birth comparing to usual care.
Cheng ^16^	Interventions for emergency contraception	Mifepristone mid-dose (25–50 mg) versus low-doses (≤ 10 mg)	Observed number of pregnancies	"Although the overall meta-analysis shows fewer pregnancies with the mid-dose... when the analysis is limited to the five trials with adequate allocation concealment...this effect is no longer evident"	Mifepristone mid-doses (25–50 mg) *may not be *more effective than low doses (≤ 10 mg)

## Discussion

The main results of this analytic survey suggest that some degree of asymmetry in funnel plots is a common finding in reproductive health meta-analyses. In about 14% of the selected meta-analyses, this type of asymmetry qualitatively impacted conclusions. However, the present study also has some limitations that have to be taken into consideration for the subject to be evaluated within context. If Cochrane meta-analyses differ in their methods from other meta-analyses or if meta-analyses with fewer than ten trials differ from those with more than ten, a selection bias may exist. Despite this possible selection bias, this source of meta-analyses was chosen because of the consistency in the methodology, associated with a widely recognized standard of quality. The number of trials was established as an inclusion criterion since this factor results in the best performance of the "trim and fill" method. Moreover, although other methods are available for the assessment of asymmetry in funnel plots, no consensus has been reached with respect to the superiority of any single method. Therefore, any method used for detecting asymmetry in funnel plots should be considered indirect and exploratory. In this study, we used the "trim and fill" method as an instrument for sensitivity analysis. Our principal concern was not the exact number of missing studies; we were, in fact, interested in how the effect size estimates would be qualitatively changed by the presence of an underlying publication bias.

Asymmetrical in funnel plots are linked to publication bias although there are other sources of asymmetry that have to be considered, including other dissemination biases, differences in the quality of smaller studies, the existence of true heterogeneity, and chance. Asymmetry in funnel plots may be an indicator that a more detailed investigation should be carried out on the presence of heterogeneity, such as sensitivity analysis.

Nevertheless, none of the meta-analyses evaluated in this study reported the use of sensitivity analysis. In the latest version of the software program generally used by Cochrane reviewers (RevMan, version 4.2.8, The Nordic Cochrane Centre, Rigshospitalet 2003), no formal test for the assessment of funnel plots is available. This software program permits the visual subjective interpretation of funnel plots, but such an approach has been shown to include a significant inter-observer variability [17]. These limitations may have restricted the use of this method in this selected sample of meta-analyses.

On the other hand, there is some concern regarding the evolution of effect size over time and the impact of including "old" trials in meta-analyses. In the past, it was possible that the determinants of data suppression and the intensity of publication bias were different when compared to those in current use. We observed that in one-third of meta-analyses, the most recent trials had been published prior to 1997 (8:21) and in more than half (11:21), the most recent trials had been published prior to 2000. In fact, it is unclear whether the date of publication would have any impact on meta-analyses in reproductive health, but caution should be taken when summarizing effects across older trials.

Rather than reviewing the conclusions of meta-analyses, the aim of this study was to provide evidence of publication bias and its consequences in selected reproductive health meta-analyses. Consequently, three examples of possible publication bias were identified. Following adjustment, a reduction in the discrepancy between the conclusions of larger trials and the conclusions of meta-analyses was seen (data not shown). In all three meta-analyses whose conclusions were not considered robust, their suggested post-adjustment conclusion was in agreement with those of the respective larger trials included. In the systematic review assessing interventions for emergency contraception [16], the meta-analysts adopted a different form of sensitivity analysis, by reanalyzing the data, and including only the trials that had adequate allocation concealment. Their findings were similar to the adjustment for funnel plot asymmetry or publication bias.

## Conclusion

Asymmetry is a frequent finding in funnel plots of meta-analyses in reproductive health, but according to the present evaluation, less than 15% of meta-analyses report conclusions that would not be considered robust. Publication bias and other sources of asymmetry in funnel plots should be systematically addressed by reproductive health meta-analysts. Next amendments in Cochrane systematic reviews should include this type of evaluation. Further studies regarding the evolution of effect size and publication bias over time in systematic reviews in reproductive health are needed.

## Competing interests

The author(s) declare that they have no competing interests.

## Authors' contributions

JPS participated in all the steps of the project, including the project development, data extraction, data analysis and writing the final report. CP participated in the project development and data extraction. JGC participated in the project development, data analysis and writing the final report. All authors provided suggestions for the manuscript, read it carefully, agreed on its content and approved the final version.

## Supplementary Material

Additional File 1 Appendix 1. Filled funnel plots of 21 reproductive health meta-analyses. Click here for file
