# Supplementary material for: Assessment of funnel plot asymmetry and publication bias in reproductive health meta-analyses: an analytic survey
Source: Reprod Health. 2007 Apr 16;4:3. doi: 10.1186/1742-4755-4-3 (PMC1855315; doi:10.1186/1742-4755-4-3)
Supplement: Additional File 1 — Appendix 1. Filled funnel plots of 21 reproductive health meta-analyses. [file 1742-4755-4-3-S1.doc]

**Publication bias on reproductive health meta-analyses: an analytical survey**

**João P Souza, Cynthia Pileggi, José G Cecatti**

**Appendix 1:**

**Filled funnel plots of 21 reproductive health meta-analyses.**

**MR1**

1. Hofmeyr GJ. Amnioinfusion for meconium-stained liquor in labour (Cochrane Review). In: *The Reproductive Health Library*, Issue 9, 2006. Oxford: Update Software Ltd. Available from http://www.rhlibrary.com. (Reprinted from *The Cochrane Library*, Issue 1, 2006. Chichester, UK: John Wiley & Sons, Ltd.)

Filled circles denoting the imputed missing studies. The bottom diamonds show summary effect estimates before (open) and after (filled) publication bias adjustment.

**MR2**

1. Smaill F, Hofmeyr GJ. Antibiotic prophylaxis for cesarean section (Cochrane Review). In: *The Reproductive Health Library*, Issue 9, 2006. Oxford: Update Software Ltd. Available from http://www.rhlibrary.com. (Reprinted from *The Cochrane Library*, Issue 1, 2006. Chichester, UK: John Wiley & Sons, Ltd.)

Filled circles denoting the imputed missing studies. The bottom diamonds show summary effect estimates before (open) and after (filled) publication bias adjustment.

**MR3**

1. Hopkins L, Smaill F. Antibiotic prophylaxis regimens and drugs for cesarean section (Cochrane Review). In: *The Reproductive Health Library*, Issue 9, 2006. Oxford: Update Software Ltd. Available from http://www.rhlibrary.com. (Reprinted from *The Cochrane Library*, Issue 1, 2006. Chichester, UK: John Wiley & Sons, Ltd.)

Filled circles denoting the imputed missing studies. The bottom diamonds show summary effect estimates before (open) and after (filled) publication bias adjustment.

**MR4**

1. Hopkins L, Smaill F. Antibiotic prophylaxis regimens and drugs for cesarean section (Cochrane Review). In: *The Reproductive Health Library*, Issue 9, 2006. Oxford: Update Software Ltd. Available from http://www.rhlibrary.com. (Reprinted from *The Cochrane Library*, Issue 1, 2006. Chichester, UK: John Wiley & Sons, Ltd.)

Filled circles denoting the imputed missing studies. The bottom diamonds show summary effect estimates before (open) and after (filled) publication bias adjustment.

**MR5**

1. Smaill F. Antibiotics for asymptomatic bacteriuria in pregnancy (Cochrane Review). In: *The Reproductive Health Library*, Issue 9, 2006. Oxford: Update Software Ltd. Available from http://www.rhlibrary.com. (Reprinted from *The Cochrane Library*, Issue 1, 2006. Chichester, UK: John Wiley & Sons, Ltd.)

Filled circles denoting the imputed missing studies. The bottom diamonds show summary effect estimates before (open) and after (filled) publication bias adjustment.

**MR6**

1. Kenyon S, Boulvain M, Neilson J. Antibiotics for preterm rupture of membranes (Cochrane Review). In: *The Reproductive Health Library*, Issue 9, 2006. Oxford: Update Software Ltd. Available from http://www.rhlibrary.com. (Reprinted from *The Cochrane Library*, Issue 1, 2006. Chichester, UK: John Wiley & Sons, Ltd.)

Filled circles denoting the imputed missing studies. The bottom diamonds show summary effect estimates before (open) and after (filled) publication bias adjustment.

**MR7**

1. McDonald H, Brocklehurst P, Parsons J. Antibiotics for treating bacterial vaginosis in pregnancy (Cochrane Review). In: *The Reproductive Health Library*, Issue 9, 2006. Oxford: Update Software Ltd. Available from http://www.rhlibrary.com. (Reprinted from *The Cochrane Library*, Issue 1, 2006. Chichester, UK: John Wiley & Sons, Ltd.)

Filled circles denoting the imputed missing studies. The bottom diamonds show summary effect estimates before (open) and after (filled) publication bias adjustment.

**MR8**

1. Abalos E, Duley L, Steyn DW, Henderson-Smart DJ. Antihypertensive drug therapy for mild to moderate hypertension during pregnancy (Cochrane Review). In: *The Reproductive Health Library*, Issue 9, 2006. Oxford: Update Software Ltd. Available from http://www.rhlibrary.com. (Reprinted from *The Cochrane Library*, Issue 1, 2006. Chichester, UK: John Wiley & Sons, Ltd.)

Filled circles denoting the imputed missing studies. The bottom diamonds show summary effect estimates before (open) and after (filled) publication bias adjustment.

**MR9**

1. Knight M, Duley L, Henderson-Smart DJ, King JF. Antiplatelet agents for preventing and treating pre-eclampsia (Cochrane Review). In: *The Reproductive Health Library*, Issue 9, 2006. Oxford: Update Software Ltd. Available from http://www.rhlibrary.com. (Reprinted from *The Cochrane Library*, Issue 1, 2006. Chichester, UK: John Wiley & Sons, Ltd.)

Filled circles denoting the imputed missing studies. The bottom diamonds show summary effect estimates before (open) and after (filled) publication bias adjustment.

**MR10**

1. King JF, Flenady VJ, Papatsonis DNM, Dekker GA, Carbonne B. Calcium channel blockers for inhibiting preterm labour (Cochrane Review). In: *The Reproductive Health Library*, Issue 9, 2006. Oxford: Update Software Ltd. Available from http://www.rhlibrary.com. (Reprinted from *The Cochrane Library*, Issue 1, 2006. Chichester, UK: John Wiley & Sons, Ltd.)

Filled circles denoting the imputed missing studies. The bottom diamonds show summary effect estimates before (open) and after (filled) publication bias adjustment.

**MR11**

1. Atallah AN, Hofmeyr GJ, Duley L. Calcium supplementation during pregnancy for preventing hypertensive disorders and related problems (Cochrane Review). In: *The Reproductive Health Library*, Issue 9, 2006. Oxford: Update Software Ltd. Available from http://www.rhlibrary.com. (Reprinted from *The Cochrane Library*, Issue 1, 2006. Chichester, UK: John Wiley & Sons, Ltd.)

Filled circles denoting the imputed missing studies. The bottom diamonds show summary effect estimates before (open) and after (filled) publication bias adjustment.

**MR12**

**(RECODED FOR ESTIMATOR <1 TO INDICATE PROTECTION OR BENEFIT)**

1. Martin-Hirsch P, Jarvis G, Kitchener H, Lilford R. Collection devices for obtaining cervical cytology samples (Cochrane Review). In: *The Reproductive Health Library*, Issue 9, 2006. Oxford: Update Software Ltd. Available from http://www.rhlibrary.com. (Reprinted from *The Cochrane Library*, Issue 1, 2006. Chichester, UK: John Wiley & Sons, Ltd.)

Filled circles denoting the imputed missing studies. The bottom diamonds show summary effect estimates before (open) and after (filled) publication bias adjustment.

**MR13**

1. Thacker SB, Stroup D, Chang M. Continuous electronic heart rate monitoring for fetal assessment during labor (Cochrane Review). In: *The Reproductive Health Library*, Issue 9, 2006. Oxford: Update Software Ltd. Available from http://www.rhlibrary.com. (Reprinted from *The Cochrane Library*, Issue 1, 2006. Chichester, UK: John Wiley & Sons, Ltd.)

Filled circles denoting the imputed missing studies. The bottom diamonds show summary effect estimates before (open) and after (filled) publication bias adjustment.

**MR14**

1. Hodnett ED, Gates S, Hofmeyr G J, Sakala C. Continuous support for women during childbirth (Cochrane Review). In: *The Reproductive Health Library*, Issue 9, 2006. Oxford: Update Software Ltd. Available from http://www.rhlibrary.com. (Reprinted from *The Cochrane Library*, Issue 1, 2006. Chichester, UK: John Wiley & Sons, Ltd.)

Filled circles denoting the imputed missing studies. The bottom diamonds show summary effect estimates before (open) and after (filled) publication bias adjustment.

**MR15**

1. Cheng L, Gülmezoglu AM, Van Oel CJ, Piaggio G, Ezcurra E, Van Look PFA. Interventions for emergency contraception (Cochrane Review). In: *The Reproductive Health Library*, Issue 9, 2006. Oxford: Update Software Ltd. Available from http://www.rhlibrary.com. (Reprinted from *The Cochrane Library*, Issue 1, 2006. Chichester, UK: John Wiley & Sons, Ltd.)

Filled circles denoting the imputed missing studies. The bottom diamonds show summary effect estimates before (open) and after (filled) publication bias adjustment.

**MR16**

1. Crowley P. Interventions for preventing or improving the outcome of delivery at or beyond term (Cochrane Review). In: *The Reproductive Health Library*, Issue 9, 2006. Oxford: Update Software Ltd. Available from http://www.rhlibrary.com. (Reprinted from *The Cochrane Library*, Issue 1, 2006. Chichester, UK: John Wiley & Sons, Ltd.)

Filled circles denoting the imputed missing studies. The bottom diamonds show summary effect estimates before (open) and after (filled) publication bias adjustment.

**MR17**

1. Oates-Whitehead RM, Haas DM, Carrier JAK. Progestogen for preventing miscarriage (Cochrane Review). In: *The Reproductive Health Library*, Issue 9, 2006. Oxford: Update Software Ltd. Available from http://www.rhlibrary.com. (Reprinted from *The Cochrane Library*, Issue 1, 2006. Chichester, UK: John Wiley & Sons, Ltd.)

Filled circles denoting the imputed missing studies. The bottom diamonds show summary effect estimates before (open) and after (filled) publication bias adjustment.

**MR18**

1. Hodnett ED, Fredericks S. Support during pregnancy for women at increased risk of low birthweight babies (Cochrane Review). In: *The Reproductive Health Library*, Issue 9, 2006. Oxford: Update Software Ltd. Available from http://www.rhlibrary.com. (Reprinted from *The Cochrane Library*, Issue 1, 2006. Chichester, UK: John Wiley & Sons, Ltd.)

Filled circles denoting the imputed missing studies. The bottom diamonds show summary effect estimates before (open) and after (filled) publication bias adjustment.

**MR19**

1. Carroli G, Bergel E. Umbilical vein injection for management of retained placenta (Cochrane Review). In: *The Reproductive Health Library*, Issue 9, 2006. Oxford: Update Software Ltd. Available from http://www.rhlibrary.com. (Reprinted from *The Cochrane Library*, Issue 1, 2006. Chichester, UK: John Wiley & Sons, Ltd.)

Filled circles denoting the imputed missing studies. The bottom diamonds show summary effect estimates before (open) and after (filled) publication bias adjustment.

**MR20**

1. Johanson RB, Menon V. Vacuum extraction versus forceps for assisted vaginal delivery (Cochrane Review). In: *The Reproductive Health Library*, Issue 9, 2006. Oxford: Update Software Ltd. Available from http://www.rhlibrary.com. (Reprinted from *The Cochrane Library*, Issue 1, 2006. Chichester, UK: John Wiley & Sons, Ltd.)

Filled circles denoting the imputed missing studies. The bottom diamonds show summary effect estimates before (open) and after (filled) publication bias adjustment.

**MR21**

1. Hofmeyr GJ , Gülmezoglu AM. Vaginal misoprostol for cervical ripening and induction of labour (Cochrane Review). In: *The Reproductive Health Library*, Issue 9, 2006. Oxford: Update Software Ltd. Available from http://www.rhlibrary.com. (Reprinted from *The Cochrane Library*, Issue 1, 2006. Chichester, UK: John Wiley & Sons, Ltd.)

Filled circles denoting the imputed missing studies. The bottom diamonds show summary effect estimates before (open) and after (filled) publication bias adjustment.
